# LDH Isoenzyme and GAA-BSA Nanoparticles: A Novel Therapy Approach for Proneural Subtype Glioblastoma Multiforme

**DOI:** 10.7150/jca.98452

**Published:** 2025-01-06

**Authors:** Mengting Yang, Xiu Han, Hongxu Li, Fengyi Du, Chunlai Feng, Aihua Gong

**Affiliations:** 1 Hematological Disease Institute of Jiangsu University, Affiliated Hospital of Jiangsu University, Zhenjiang, China, 212001.; 2 Center of Clinical Laboratory, Dushu Lake Hospital Affiliated to Soochow University, Medical Center of Soochow University, Suzhou Dushu Lake Hospital, Suzhou, Jiangsu, China, 215213.; 3 Department of Pharmaceutics, School of Pharmacy, Jiangsu University, Zhenjiang, China.

**Keywords:** glioblastoma multiform, lactate dehydrogenase, subtype classification, proneural-to-mesenchymal transition, nanoparticles

## Abstract

Glioblastoma multiforme (GBM), whose pathogenesis involves proneural-to-mesenchymal transition (PMT), is the most malignant type of glioma and is associated with a bleak prognosis. Lactate dehydrogenase (LDH) comprises two major subunits, LDHA and LDHB, which can assemble into five different isoenzymes (LDH1-5). However, the role of LDH isoenzyme and its subunits in different GBM subtypes is largely unknown. Our findings reveal that LDHA and LDHB subunits correlated with mesenchymal and proneural subtype classification, and have prognostic and clinical significance in GBM patients. Moreover, it is demonstrated that LDH5, characterized by high LDHA and low LDHB levels, is highly expressed in mesenchymal subtype GBM cells and promotes proliferation, migration, and PMT. Conversely, proneural subtype GBM cells exhibited LDH1 dominance, and low LDHA and high LDHB levels. Notably, LDH1 played a pivotal role in the proliferation, migration, and PMT of proneural glioma cells. For treatment of proneural subtype GBM, gossypol-acetic acid (GAA)-bovine serum albumin (BSA) nanoparticles (GAA-BSA NPs) were developed to ameliorate PMT by targeting LDH1. These nanoparticles effectively suppress proneural subtype tumor growth both *in vitro* and *in vivo*, surpassing their efficacy against the mesenchymal subtype. The results offer several novel insights into the role of LDH isoenzyme in subtype classification between mesenchymal and proneural GBM and provide a promising therapeutic approach for proneural subtype GBM.

## Introduction

Glioblastoma multiforme (GBM), classified as a World Health Organization grade IV tumor, is extremely malignant and has a high prevalence rate, with a grim clinical prognosis among adults 1. Despite the availability of comprehensive treatment strategies encompassing surgical intervention, ionizing radiation, and chemotherapy with the alkylating agent temozolomide, the median survival of patients with GBM following diagnosis rarely exceeds 1-year 2, 3. Distinguished by their gene expression profiles, GBMs can be categorized into four distinct subtypes, namely, classical, proneural, neural, and mesenchymal 4, 5. During the progression of GBM, the mesenchymal subtype originates from the proneural subtype through a process known as proneural-to-mesenchymal transition (PMT) 6. This transition is associated with tumor microenvironment, metabolic molecular and treatment-related factors to tumor recurrent and therapy 7.

Lactate dehydrogenase (LDH) is the core enzyme involved in catalyzing the formation of lactate through glycolysis; this process, known as the Warburg effect, is a hallmark of metabolic alteration in cancer cells 8. The LDH isoenzyme is composed of two subunits (LDHA and LDHB), forming a tetramer structure that produces five key isoforms, namely, LDH1(4B), LDH2(3B1A), LDH3(2B2A), LDH4(3B1A), and LDH5(4A) 9, 10. LDH isoenzyme shows variations in distribution among various tissues in the human body and between malignant tissue samples, indicating it can serve as a vital molecular marker for cancer diagnosis 11. Although the effects of LDH isoenzyme on GBM growth and invasiveness have been studied 12, its role and potential as a therapeutic target in PMT warrants further investigation.

Although many effective LDHA/B inhibitors that target LDH isoenzyme have been developed by pharmaceutical companies and academic institutions, these inhibitors have not been applied in the clinic for various reasons such as poor pharmacokinetic properties and low *in vivo* efficacy 13, 14. Gossypol-acetic acid (GAA), a dehydrogenase inhibitor, has demonstrated the capacity to interfere with mitochondrial metabolism, induce autophagic cell death, and promote tumor cell apoptosis 15. However, clinical studies in patients with glioma have shown a low response rate to treatment with GAA 16, although the reasons responsible for this outcome remain unclear. Unique tumor microenvironment characterized by hypoxia and high ferric ions (Fe^3+^) levels features often encountered in gliomas, and iron chelators can change the metabolism of tumor cells by reducing the intake of Fe^3+^ 17, 18. Studies have shown that GAA tends to bind with iron in hemoglobin to form gossypol-iron complexes, which in turn inhibit iron absorption^19^. Within this context, we hypothesized that tumor microenvironment's high Fe^3+^ levels may influence the efficacy of GAA in tumor treatment. Owing to its exceptional biocompatibility and biodegradability, bovine serum albumin (BSA) is commonly utilized as a drug carrier 20. Additionally, BSA nanoparticles (NPs) are soluble in water, stable, and capable of gradually releasing hydrophobic drugs 21. These attributes render them promising tools for the treatment of GBM 22.

In the present study, we investigated the different roles of the LDH isoenzyme (LDH1 and LDH5) within the proneural and mesenchymal GBM subtypes, respectively, and examined their key roles in the PMT process. Furthermore, we developed a strategy targeting LDH1 through encapsulating GAA with BSA to generate NPs that could be used in the treatment of proneural subtype GBM. Our results offer novel insights into the role of LDH isoenzyme in subtype classification between mesenchymal and proneural GBM and provide a promising treatment approach for proneural subtype GBM.

## Materials and methods

### Data source

RNA-seq transcriptome data and clinical data (survival time and survival status) of patients with glioblastoma were collected from The Cancer Genome Atlas (TCGA) database (http://cancergenome.nih.gov/) and The Chinese Glioma Genome Atlas (CGGA) database (http://www.cgga.org.cn/). Verhaak glioblastoma mesenchymal and proneural gene signatures were obtained from the Molecular Signatures Database (https://www.gsea-msigdb.org/gsea/index.jsp). The TCGA data set downloaded was consisting of 599 cases, with 369 males and 230 females. Among these, primary clinical data (including age, pathology, and survival) is available for 596 cases, while secondary clinical data (including treatment information) is available for 550 cases. The data in the TCGA database mainly comprises primary GBM cases, where primary GBM patients have no antecedent history of lower-grade disease, whereas secondary GBM arises from previously diagnosed lower-grade gliomas^23^. The samples of Verhaak database were collected and processed through the TCGA Biospecimens Core Resource, 200 GBMs and 2 normal samples were selected within specific standards^24^. All the subjects of CGGA were consistently diagnosed with glioma and were then further classified according to the 2007/2016 WHO classification system, clinical data, including basic clinical information, survival, and therapy information were available^25^.

### Survival analysis

Kaplan-Meier plots were used to assess the relationship between LDHA and LDHB expression and prognosis (OS) of cancers. Proportional-hazards hypothesis testing and fitted survival regression were performed with the survival package (version 3.3.1), and the results were visualized with the survminer package and the ggplot2 (version 3.3.6) package. The Log-rank test was used in the hypothesis test, and p < 0.05 is considered statistically significant.

### Clinical significance and prognosis analysis

Forest map was used for further clinical significance analysis of LDH in GBM. The survival package (version 3.3.1) was used for proportional hazards hypothesis testing and Cox regression analysis. The survival package was used for proportional hazards hypothesis testing and Cox regression analysis. Forest map visualization was performed using ggplot2 (version 3.3.6).

### Cell lines and cell culture

The human glioma cell lines SW1783, LN229, U87MG, and U251MG were obtained from the American Type Culture Collection (ATCC, Manassas, VA, USA). The cells were cultured at 37°C under normoxic conditions (21% O_2_ and 5% CO_2_) or hypoxic conditions (1% O_2_, 5% CO_2_, and 94% N_2_), and maintained in Dulbecco's modified Eagle's medium (DMEM; Meilun, Dalian, China) supplemented with 10% fetal bovine serum (FBS; Gibco, Carlsbad, CA, USA) for up to 3 months after resuscitation from frozen stocks, with fewer than 20 passages. Short tandem repeat profiling was used to authenticate the cell lines.

### Plasmid construction, transfection

The short hairpin sh-EGFP, sh-LDHA, and sh-LDHB plasmids were previously constructed in our laboratory. The p3×FLAG Myc-CMV™14 expression vector and pLKO.1-puro or pLKOTet-ON-puro vectors were purchased from Sigma-Aldrich (San Francisco, CA, USA). Cells were seeded in six-well plates to reach 70-90% confluency before transfection. The next day, transfection was performed according to the instructions provided by the manufacturer, utilizing Lipofectamine® 2000 transfection reagent (Invitrogen, Carlsbad, CA, USA). Following an additional 48 h of incubation, the cells were analyzed using various techniques described below. Each test was repeated in triplicate.

### RNA extraction, reverse transcription, and quantitative real-time PCR

Total RNA was extracted using RNAiso Plus (Vazyme, Nanjing, China). Reverse transcription was performed using a RevertAid First Strand cDNA Synthesis Kit (Vazyme, Nanjing, China) according to the instructions provided by the manufacturer. Quantitative real-time PCR was performed in triplicate in 20 μL reactions with a SYBR GREEN PCR Kit (Vazyme, Nanjing, China). The 2^-ΔΔCT^ method, with β-actin serving as an endogenous control, was used to calculate the relative mRNA expression. The sequences of primers used are provided in Table 1. All experiments were conducted in biological triplicates.

### Western blotting analysis

Before treatment with radioimmunoprecipitation assay lysis buffer at 4°C for 10 min, the cultured cells were rinsed with cold phosphate-buffered saline (PBS). The mixture was then heated at 100°C for 10 min and centrifuged at 4°C at 12,000 × *g* min-1 for 10 min. The obtained total protein was subjected to 10% sodium dodecyl sulfate-polyacrylamide gel electrophoresis (~20 μg of protein per lane). Subsequently, the proteins were transferred onto a polyvinylidene difluoride membrane, which was blocked with 5% BSA for 1 h at room temperature. Thereafter, the membrane was incubated with primary antibodies overnight at 4°C, followed by incubation at 1 h at room temperature with secondary antibodies. The following primary antibodies were used at dilutions recommended by the manufacturers: anti-LDHA (sc-137243; SCBT, Santa Cruz, CA, USA), anti-LDHB (sc-100775; SCBT, Santa Cruz, CA, USA), anti-oligodendrocyte transcription factor 2 (anti-OLIG2; sc-293163; SCBT, Santa Cruz, CA, USA), anti-vimentin (anti-VIM; 5741; Cell Signaling Technology, Danvers, MA, USA), and anti-β-Tubulin (MA5-11732; Thermo Fisher Scientific, MA, USA). Goat anti-rabbit (sc-2004; SCBT, Santa Cruz, CA, USA) and goat anti-mouse secondary antibodies (sc-2005; SCBT, Santa Cruz, CA, USA) conjugated with horseradish peroxidase were used for detection. Visualization was performed using an enhanced chemiluminescence substrate. β-Tubulin served as a loading control. All experiments were conducted in biological triplicates. Protein expression was quantitatively analyzed using ImageJ software (National Institutes of Health, Bethesda, MD, USA).

### Native gel electrophoresis for the separation of LDH isoenzymes

Separation of LDH isoenzymes through native gel electrophoresis was conducted using precast 6.5% polyacrylamide slab gels. Before treatment with radioimmunoprecipitation assay lysis buffer at 4°C for 30 min, cultured cells and mouse brain tissues were rinsed with cold PBS. The sample wells were rinsed with running buffer, and the gels were positioned in the electrophoretic unit. Subsequently, the gel was placed in a developing chamber containing the developer solution (H_2_O 18.4 mL, 1 M Tris 4 mL, tetrazolium-blue 12 mL, phenazine methosulphate 4 mL, Na-lactate 4 mL, and nicotinamide adenine dinucleotide [NAD] 1.3 mL) followed by incubation at 37°C for 30 min to initiate a color reaction. Finally, the gel was rinsed with water.

### Cell Counting Kit-8 (CCK-8) assay

The measurement of viable cell mass was performed using a CCK-8 (Vazyme) according to the instructions provided by the manufacturer. Briefly, cells were seeded in a 96-well plate (1×10^3^ cells/well) and incubated at 37°C with 5% CO_2_. On days 1-6, CCK-8 solution (10 μL) was added to each well, and cells were incubated at 37°C for 2 h. Next, the absorbance was determined at 450 nm wavelength. Cytotoxicity was evaluated by CCK-8 assay according to the instructions provided by the manufacturer. Briefly, cells were seeded in 96-well flat-bottomed plates (5×10^3^ cells/well) and subjected to the following treatments: 1) 50 μmol/L 0.2 M in 2-methyl tetrahydrofuran (Fecl_3_; 710857; Sigma-Aldrich), 2) different concentrations of GAA (A506218-0001; Sangon Biotech, Shanghai, China), and 3) different concentrations of GAA-BSA NPs.

### Colony formation assay

Transfected glioma cells were harvested and resuspended in medium. Next, cells were transferred to a six-well plate (500, 1×10^3^, and 2×10^3^ cells per well) for 10-14 days until large colonies were visible. Colonies were fixed and stained with 0.05% crystal violet for 30 min. The number of colonies was counted.

### Transwell migration assays

Transfected cells were collected, resuspended in serum-free medium, and transferred to Transwell upper chamber with permeable inserts featuring membranes with 8 μm pores (1×10^4^ cells per well). The lower chamber was filled with culture medium with 10% FBS for 10 h. The cells on the upper chamber were scraped and washed with PBS, and stained with 4% paraformaldehyde for 30 min. Finally, cells were counted in five independent fields and the average number of cells per field was used to produce the graphs. Each test was repeated in triplicate.

### Tissue microarray immunohistochemistry

Tissue sections were air-dried, deparaffinized, and rehydrated. Endogenous peroxidase activity was blocked with H_2_O_2_ (3%) for 10 min. Antigen retrieval was performed with citrate buffer in a pressure cooker for 10 min. A 5% serum-free protein block was applied for 20 min to block non-specific antibody binding. Subsequently, slides were incubated overnight at 4°C with the primary antibody anti-LDHA (sc-137243; SCBT, Santa Cruz, CA, USA), anti-LDHB (sc-100775; SCBT, Santa Cruz, CA, USA), anti-YKL-40 (BS6564; Bioworld Technology, Bloomington, USA) and anti-vimentin (anti-VIM; 5741; Cell Signaling Technology, Danvers, MA, USA), followed by incubation at room temperature for 30 min with a species-specific secondary antibody (SA1020; BOSTER, Wuhan, China). For negative controls, the primary antibody was omitted and replaced with negative immunoglobulin G. The reaction was developed using 3,3'-diaminobenzidine (AR1022; BOSTER, Wuhan, China), and slides were counterstained with hematoxylin. Immunostaining intensity and reactivity were assessed using CaseViewer after scanning, employing a digital microscope application at ×40 magnification.

The tissue microarray was procured from Easy Biotrade (Nanjing, China). Human glioma samples employed in this study were validated by a pathologist based on the World Health Organization criteria. This tissue microarray consists of 60 clinical samples, all of which were glioblastoma (Grade IV glioma) cases. Among these, 39 were male and 21 were female, with an age range of 20 to 78 years. A heatmap was generated from these data, and conditional formatting in MS Excel (Microsoft Corp., Redmond, WA, USA) was used to produce colored heatmaps.

### Molecular docking and site prediction

The three-dimensional structure of LDH1 was evaluated according to the probability density function or Discrete Optimized Protein Energy fraction of the statistical potential energy of atoms. Based on the optimal three-dimensional structure of LDH1, Glide software for molecular docking was used to screen small-molecule compounds from databases (such as ChemDiv, ChemBridge, and SPECS) with different precision scoring functions (high-throughput virtual screening, standard precision, and extra precision) and analyze the binding sites.

### Synthesis of GAA-BSA NPs

GAA-BSA NPs were synthesized using the previously reported desolvation method 26. Briefly, 30 mg of BSA (4240GR100; BioFroxx Hangzhou, China) in 1 mL of pure water was adjusted to pH 8.2 using sodium hydroxide (NaOH; DWH750; Hushi, Shanghai, China). Next, we dissolved 1 mg of GAA (A506218-0001; Sangon Biotech) in 3 mL of ethanol and added it dropwise to the BSA solution at a rate of 1 mL min^-1^ while stirring using a magnetic stirrer at 700 rpm and a temperature of 37°C. The NPs were formed after stirring for 12 h, during which 8% glutaraldehyde solution was added for cross-linking. The NP suspension was purified by ultracentrifugation at 12,000 rpm for 30 min, and subsequently redispersed in pure water under sonication. Finally, GAA-BSA NPs were obtained by freeze-drying.

### Size distribution, polydispersity, and zeta potential measurement

The particle size, polydispersity index, and zeta potential were determined at 25°C using a Zetasizer (Nano ZS; Malvern, UK). All measurements were performed in triplicate.

### Morphological analysis

For morphological study, the GAA-BSA NP powder was evenly scattered on the sample table with a toothpick and sprayed with gold. Subsequently, the NPs were observed using a scanning electron microscope (Scio2 HiVac; FEI, USA). To further investigate the internal structure of GAA-BSA NPs, the suspension was dropped on a copper grating and the samples were observed using a transmission electron microscope (Tecnai 12; FEI).

### Determination of drug-loading efficiency (DLE) and drug-loading capacity (DLC)

To calculate the DLE and DLC, 1.003 mg of GAA-BSA NP powder was precisely weighed, dissolved in 1 mL of pure water, and made up to a volume of 10 mL with methanol. After stirring and centrifugation, the content of GAA in the supernatant was measured using high-performance liquid chromatography. The analysis was performed on a VP-ODS C18 column (250 mm×4.6 mm×5 µm; SHIMADZU, Tokyo, Japan) at 35°C. The mobile phase was a mixture of potassium dihydrogen phosphate and acetonitrile in a volume ratio of 50:50 (volume/volume). The detection wavelength was set at 247 nm. DLE and DLC were calculated using the following equations:

DLE = 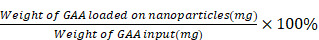


DLC = 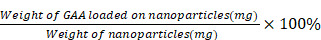


### In vitro drug release tests

*In vitro* release of GAA-BSA NPs was studied using a dynamic dialysis method. Specifically, GAA-BSA NPs (containing 0.2 mg of GAA) and 0.2 mg GAA were dissolved in 1 mL of PBS (with 3% dimethyl sulfoxide) and transferred to dialysis bags with a molecular weight cut-off of 3,500. Next, the bags were submerged in 50 mL of 0.1 M PBS (pH 7.4) and maintained at 37°C with shaking at a speed of 120 rpm. At predetermined time intervals (from 30 min to 96 h), 1 mL of the medium was withdrawn and replenished with an equal volume of fresh medium. The samples were diluted with methanol and subjected to high-performance liquid chromatography analysis after centrifugation. Each measurement was performed in triplicate. Animal studies were approved by the Ethics Committee of Jiangsu University (approval number: 2021102901) and complied with the ARRIVE guidelines and the National Research Council's Guide for the Care and Use of Laboratory Animals.

### Cellular binding and internalization

The GAA-BSA NPs were labelled with fluorescein isothiocyanate (FITC; F7250; Merck, Darmstadt, Germany; excitation wavelength: 488 nm, emission wavelength: 517 nm) (GAA-BSA-FITC NPs). U87MG and U251MG cells (0.5 mL, 1 × 10^5^ cells mL^-1^) were seeded in a confocal dish and incubated in 1 mL of DMEM containing 10% FBS for 12 h. Thereafter, 100 μg mL^-1^ of GAA-BSA-FITC NPs were added into the culture medium and co-cultured with cells at 37°C for 4 h. Following incubation, the cells were fixed with 4% paraformaldehyde for 15 min. Thereafter, the cells were washed three times with PBS and stained with 4,6-diamidino-2-phenylindole (DAPI; 10236276001; Merck) at room temperature. The binding and internalization behavior of the nanoplatforms within the cells were observed using a confocal microscope (LSM710; Zeiss, Oberkochen, Germany).

### Tumor-bearing mouse model

Nude mice (males, 4-6 weeks old) were purchased from CAVENS company (Changzhou, China). Mice were housed under pathogen-free conditions with free access to food and water, at the Animal Center of Jiangsu University (Zhenjiang, China). Animal studies were approved by the Ethics Committee of Jiangsu University (approval number: 2021102901) and complied with the ARRIVE guidelines and National Research Council's Guide for the Care and Use of Laboratory Animals. The mice received an injection of 0.1 mL of U87MG cells (2×10^5^) transfected with sh-EGFP, sh-LDHA, and sh-LDHB plasmids. In addition, the mice received an injection of 0.1 mL of U251MG cells (8×10^5^) transfected with sh-EGFP, sh-LDHA, and sh-LDHB plasmids. Furthermore, the other U87MG- and U251MG-bearing mouse models were established by subcutaneously injecting 0.1 mL of U87MG cells (2×10^5^) and U251MG cells (8×10^5^), respectively, into the BALB/c nude mice. When the tumor volume reached approximately 80 mm^3^, the tumor-bearing mice were randomized into four groups (n=6 per group). Mice in different groups were administered intraperitoneal injections once every 3 days, for six times in total, as follows: 1) PBS; 2) GAA, 15 mg/kg; 3) GAA-BSA NPs at 15 mg/kg GAA dose; and 4) LDHA inhibitor FX11 (2.5 mg/kg, HY16214; MedChemExpress, Shanghai, China). Tumor size was monitored throughout the treatment period.

### Statistical analysis

All statistical analyses were conducted using GraphPad Prism 9 (GraphPad Software Inc., San Diego, CA, USA). Student's *t*-test was used for comparisons between two groups, and one-way ANOVA analysis of variance was utilized for comparisons involving more than two groups. Data are presented as the mean ± standard deviation of three independent experiments. P-values of <0.05 indicate statistically significant differences (*P value < 0.05; **P value < 0.01; ***P value < 0.001).

## Results

### LDH subunits are correlated with mesenchymal and proneural subtype classification and have clinical significance in GBM patients

**Figure 1:**
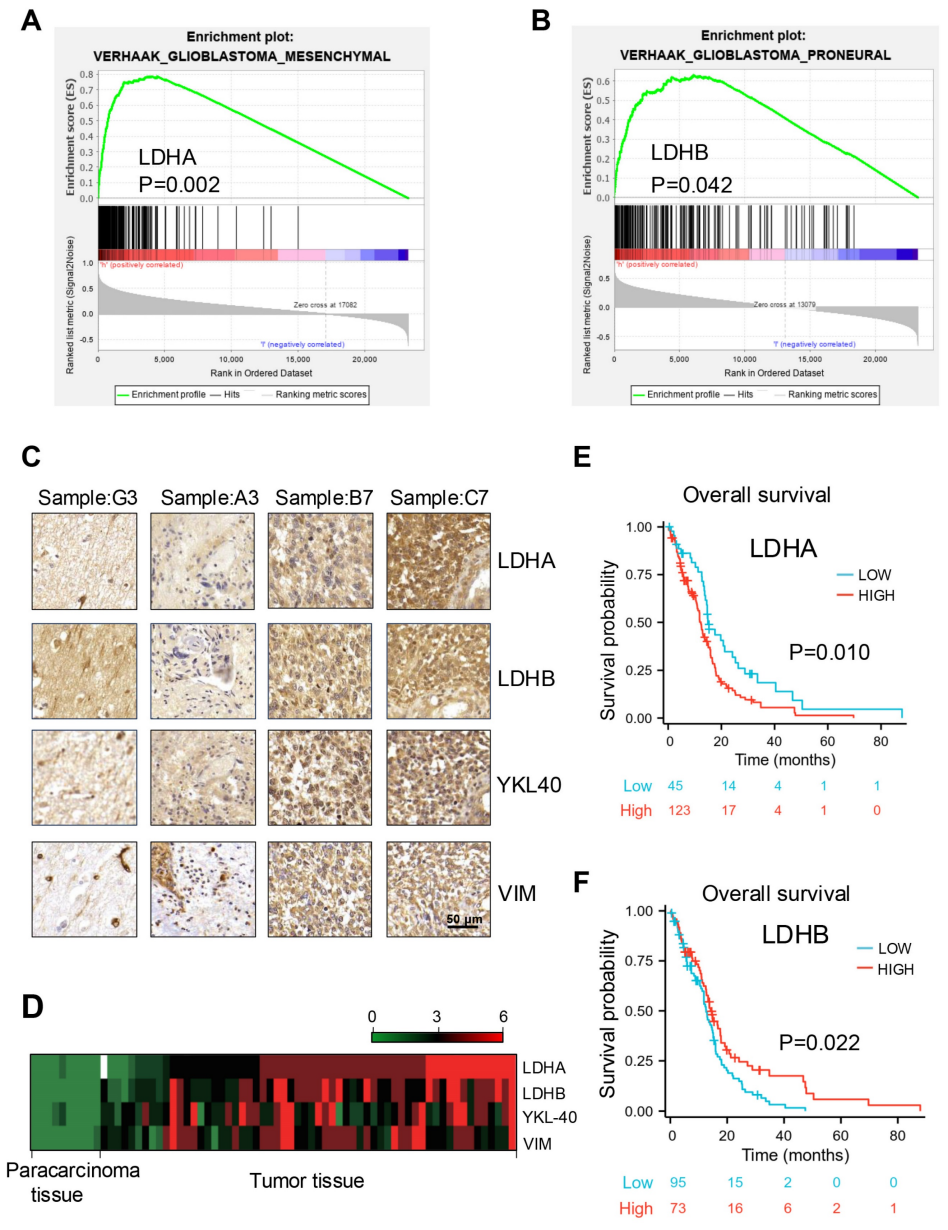
Expression profiles of LDH subunits and their prognostic significance in GBM. (**A, B**) GSEA based on the Verhaak gene dataset indicated that LDHA and LDHB expression was positively correlated with the mesenchymal (A) and proneural (B) subtype, respectively (P=0.002 and 0.042, respectively). (**C**) Immunohistochemical detection of protein expression levels of LDHA, LDHB, YKL-40, and VIM in human GBM samples. Scale bar: 50 μm. (**D**) Heatmap analysis presenting the expression of LDHA, LDHB, YKL-40, and VIM in human GBM samples, based on the results obtained from immunohistochemistry. (**E, F**) Association of LDHA (**E**) and LDHB (**F**) expression with overall survival analysis of patients with GBM (P=0.010 and 0.022, respectively). P-values were determined by one-way ANOVA.

Initially, the relationship between LDH subunits and mesenchymal and proneural molecular subtypes was identified through gene set enrichment analysis (GSEA) using Verhaak gene datasets. The results demonstrated that elevated levels of LDHA were predominantly observed in the mesenchymal subtype, whereas reduced LDHA expression was more frequently associated with the proneural subtype. (Fig. 1A and S1A). Conversely, the expression levels of LDHB exhibited an opposite pattern. (Fig. 1B and S1B). Further, a positive correlation was detected between the expression of mesenchymal markers (YKL-40 and VIM) and LDHA levels by immunohistochemical analysis (Fig. 1C). These findings were further validated by heatmap analysis (Fig. 1D). In addition, we investigated the impact of LDHA and LDHB on GBM prognosis through Kaplan-Meier survival analysis using The Cancer Genome Atlas (TCGA) mRNA database. The results indicated that patients exhibiting high LDHA expression experienced shorter survival periods, while elevated LDHB expression appears to be associated with prolonged survival periods (Fig. 1E and F). After a comprehensive evaluation of histology, grade, gender, and age, univariate and multivariate Cox regression analyses were employed using the Chinese Glioma Genome Atlas (CGGA) database to identify prognostic factors and examine their clinical relevance. (Fig. S1 C-F). These studies identified LDHA and LDHB as potential independent overall survival factors, indicating their potential utility in constructing prognostic calibration curves for predicting GBM outcomes and underscoring their significant clinical relevance.

### LDH isoenzymes composed of different subunits determine the classification of glioma cells

**Figure 2:**
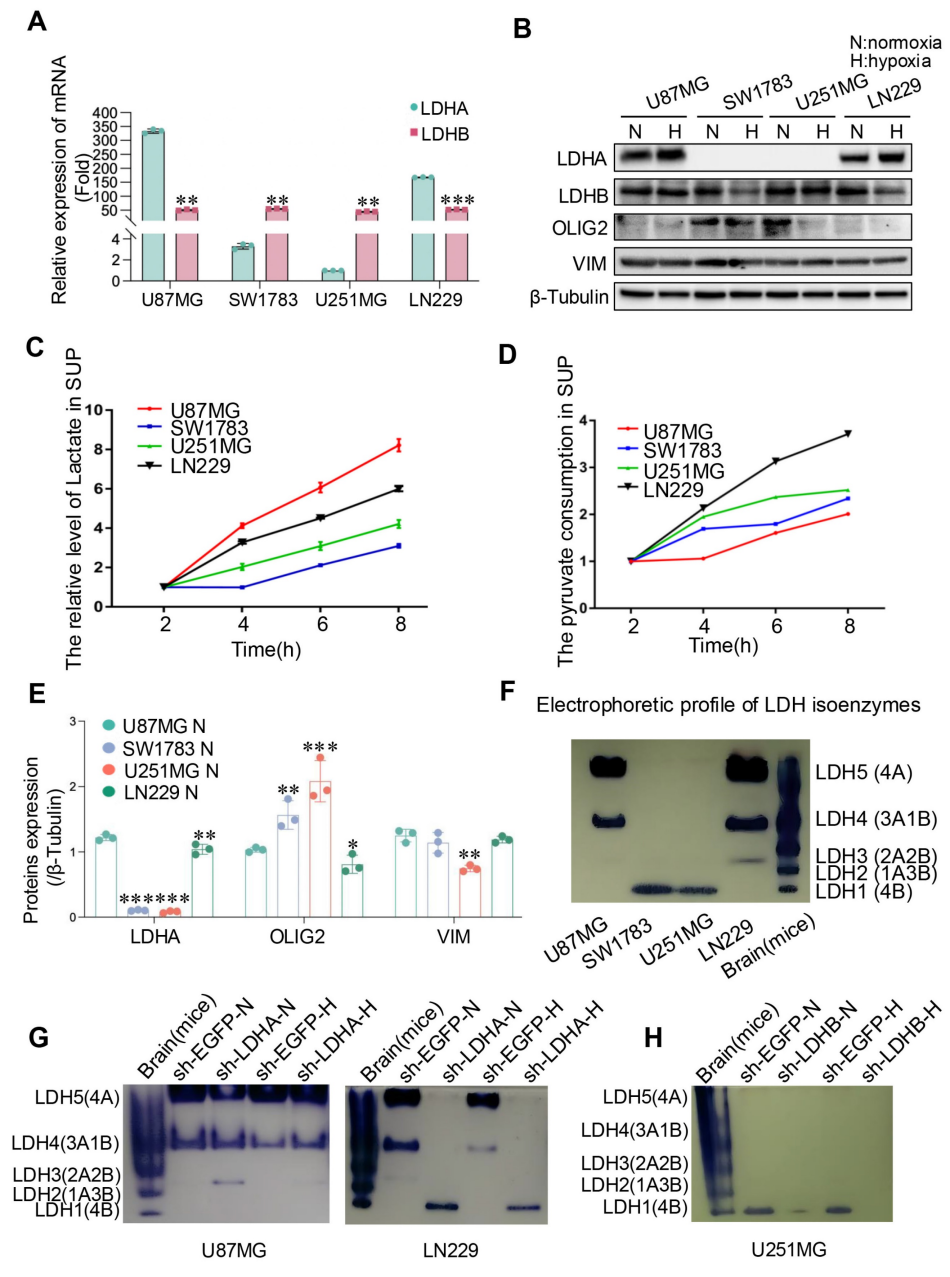
The expression of LDH isoenzymes in human glioma cells. (**A, B**) The mRNA (A) and protein (B) expression levels of LDHA and LDHB in human glioma cells were quantified through qRT-PCR and western blotting, respectively. N, normoxic condition; H, hypoxic condition. (**C, D**) Lactate and pyruvate levels were measured in four human glioma cell lines through spectrophotometry. (**E**) Quantitative analysis of protein expression levels in (B) was conducted using ImageJ software. (**F, G, H**) LDH isoenzyme of glioma cells were analyzed using the native gel electrophoresis method, illustrating the normal pattern (F), alterations resulting from LDHA or LDHB knockdown in U87MG, LN229 (G), and U251MG (H) cells. Statistical analysis was conducted using one-way ANOVA; *P<0.05, **P<0.01, and ***P<0.001.

To validate our database-derived analyses, we conducted in vitro experiments across four glioma cell lines. Initially, LDHA and LDHB expression levels were tested. Results indicated a marked upregulation of LDHA at both mRNA and protein levels in LN229 and U87MG cells, whereas LDHB was significantly expressed in U251MG and SW1783 cells at both mRNA and protein levels (Fig. 2A and B), highlighting differential LDH subunit expression across these glioma cell lines.

Considering the pivotal role of LDH in lactate production, we quantified lactate concentrations in the cell culture supernatants and assessed the dynamics of pyruvate consumption. Observations revealed that U87MG and LN229 glioma cells exhibited higher lactate production rates compared to SW1783 and U251MG cells. Notably, U87MG cells produced substantial amounts of lactate while consuming relatively low levels of pyruvate, suggesting an enhanced pyruvate utilization rate (Fig. 2C and D), which may due to variations in their metabolic pathways and enzyme activity^27^-^29^. To further elucidate the association between LDH subunits and glioblastoma (GBM) phenotypes, we analyzed the expression of OLIG2 (a marker for the proneural phenotype) and VIM (a marker for the mesenchymal phenotype). Consistent with previously observed patterns in GBM specimens, OLIG2 expression was elevated in SW1783 and U251MG cells, while U87MG and LN229 cells exhibited characteristics associated with mesenchymal glioma cells (Fig. 2B and E).

Additionally, native electrophoresis corroborated these findings, revealing that distinct LDH isoenzyme expression profiles were present across the cell lines due to variations in LDH subunit expression. Specifically, LDH5 (4A) was the predominant isoform in LN229 and U87MG cells, whereas LDH1 (4B) was the main isoform in U251MG and SW1783 cells (Fig. 2F). To confirm that the observed isoenzyme patterns were attributable to differential LDH subunit expression, we selectively suppressed LDHA and LDHB using shRNA constructs (sh-LDHA and sh-LDHB, respectively). Knockdown of LDHA and LDHB led to significant reductions in their respective mRNA levels in U87MG, LN229, and U251MG cells relative to control cells (sh-EGFP) (Fig. S2A-C). Furthermore, native electrophoresis demonstrated that LDHA knockdown resulted in diminished LDH5 expression in U87MG and LN229 cells, while LDHB knockdown had minimal impact (Fig. 2G and S3A). Conversely, LDHB inhibition led to decreased LDH1 expression in U251MG cells, whereas LDHA knockdown did not alter the LDH isoenzyme profile (Fig. 2H and S3B). Collectively, these findings indicate that the differential expression of LDH subunits contributes to distinct LDH isoenzyme compositions, which may play a role in defining glioma cell phenotypes.

### LDH isoenzymes promoted the proliferation and migration of glioma cells and in vivo tumor growth

**Figure 3:**
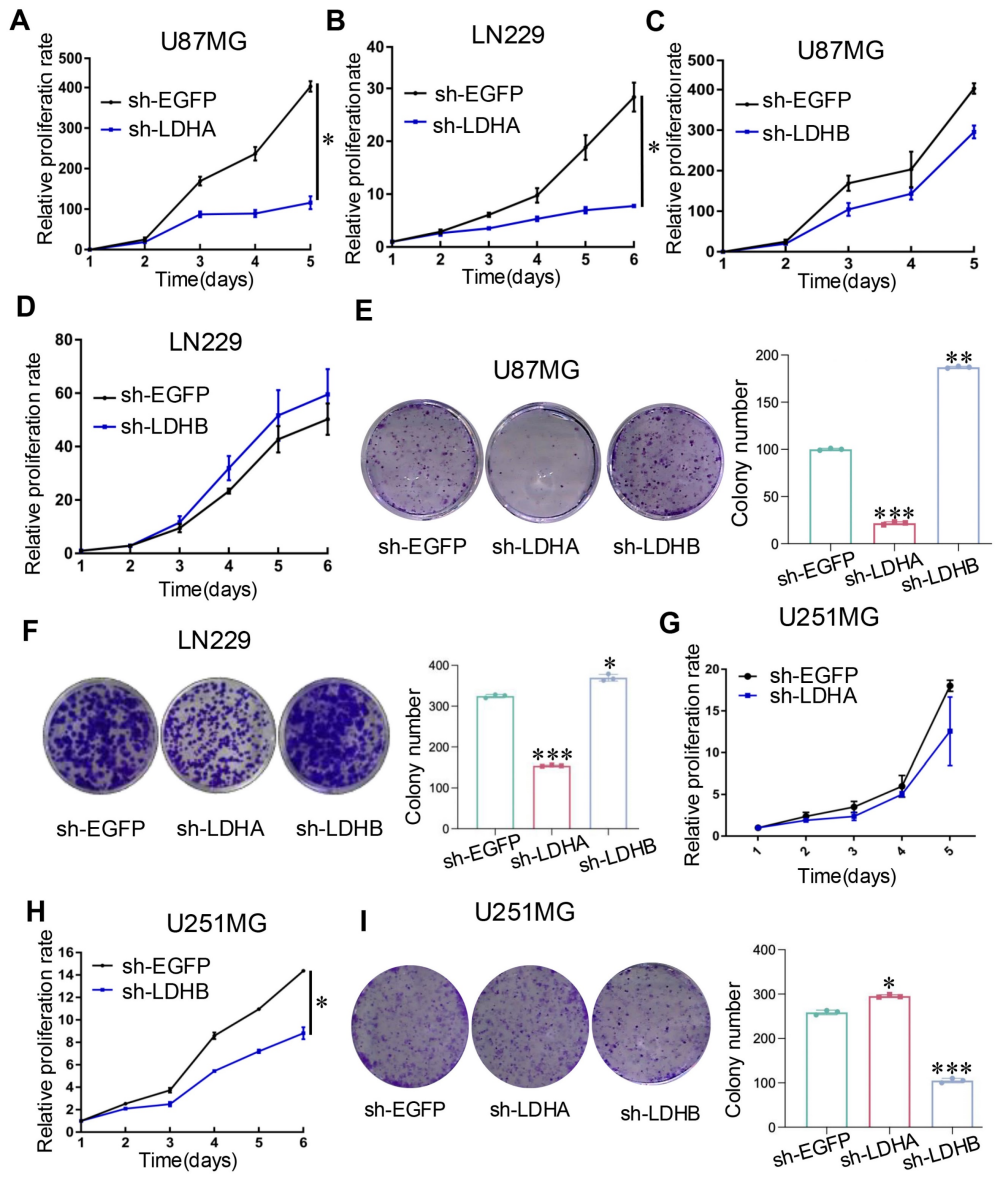
LDH isoenzymes promoted the proliferation of human glioma cells. (**A, B**) CCK-8 assay revealed the impact of LDHA knockdown relative to the control in U87MG cells (A) and LN229 cells (B). (**C, D**) CCK-8 assay revealed the impact of LDHB knockdown in U87MG cells (C) and LN229 cells (D). (**E, F**) Colony formation assay depicting differences in cell proliferation between U87MG (E) and LN229 (F) cells. (**G, H**) The proliferative ability of U251MG transfected with sh-EGFP, sh-LDHA (G), or sh-LDHB (H) plasmids determined using CCK-8 assay. (**I**) Colony formation assay depicting cell proliferation in U251MG cells. Statistical analysis was conducted using one-way ANOVA; *P<0.05, **P<0.01, and ***P<0.001.

Downregulating the expression of LDHA inhibited the relative proliferation rate of U87MG and LN229 cells in comparison with that in the control group (Fig. 3A and B). Although different outcomes in cell proliferation rates were observed in U87MG and LN229 cells when LDHB was downregulated, there was no statistically significant difference (Fig. 3C and D). The results were further confirmed by colony formation assays (Fig. 3E and F). In contrast, an inverse trend was noted in U251MG cells: LDHB downregulation led to a significant decrease in relative proliferation rate and colony numbers, whereas LDHA downregulation had the opposite effect (Fig. 3G-I).

**Figure 4:**
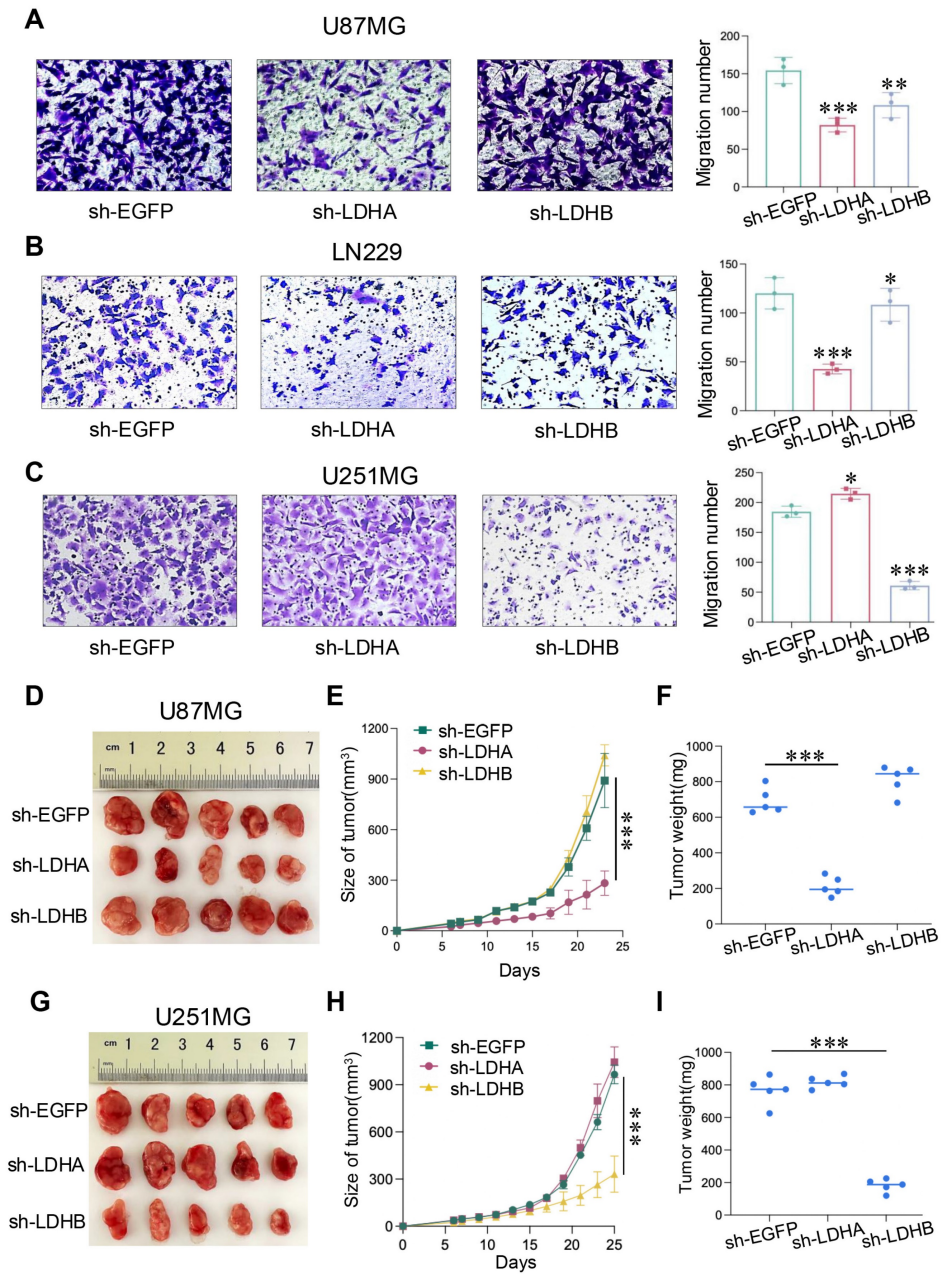
LDH isoenzymes promoted the migration of human glioma cells and *in vivo* tumor growth. (**A-C**) Transwell migration assay comparing the sh-LDHA or sh-LDHB group with the sh-EGFP group of U87MG (A), LN229 (B), and U251MG (C) cells. (**D**) Photographs showing U87MG model tumors after treatment. (**E**) Changes in U87MG tumor volume following different treatments. (**F**) Mean tumor weights of U87MG tumors. (**G**) Photographs showing U251MG tumors after treatment. (**H**) Measurement of U251MG tumor volume at different time points. (**I**) Mean tumor weights of U251MG tumors. Data are presented as the mean ± standard deviation. Statistical analysis was conducted using one-way ANOVA; *P<0.05, **P<0.01, and ***P<0.001.

We further investigated the influence of LDH isoenzymes on glioma cell migration. Notably, LDHA knockdown led to a reduction in the number of migrating U87MG and LN229 cells, as evidenced by the Transwell assay, whereas LDHB knockdown exerted the opposite effect (Fig. 4A and B). Conversely, in U251MG cells, sh-LDHB resulted in reduced migratory ability, whereas the number of migrated cells increased in the sh-LDHA group (Fig. 4C). These findings underscore the distinct and critical roles played by LDHA and LDHB in these two glioma subtypes. In summary, LDH5 promotes the proliferation and migration of mesenchymal glioma cells, whereas LDH1 maintains these processes in proneural glioma cells. Thus, LDH isoenzyme differentially regulates the proliferation and migration of mesenchymal and proneural glioma cells.

*In vivo* studies were conducted by injecting U87MG and U251MG cells into the BALB/c nude mice. Intriguingly, in U87MG group, LDHA knockdown led to decreased tumor growth, whereas LDHB knockdown exerted the opposite effect (Fig. 4D). These results were also observed in terms of tumor volume and weight (Fig. 4E and F). Conversely, in the U251MG group, LDHB knockdown significantly decreased the tumor size, volume, and weight, whereas LDHA knockdown exerted the opposite effect (Fig. 4G-I). Collectively, we found that LDH5 and LDH1 play pivotal roles in the proliferation and migration of glioma cells as well as tumor growth in mesenchymal and proneural subtypes of GBM.

### LDH isoenzymes promoted proneural-to-mesenchymal transition (PMT) of glioma cells

**Figure 5:**
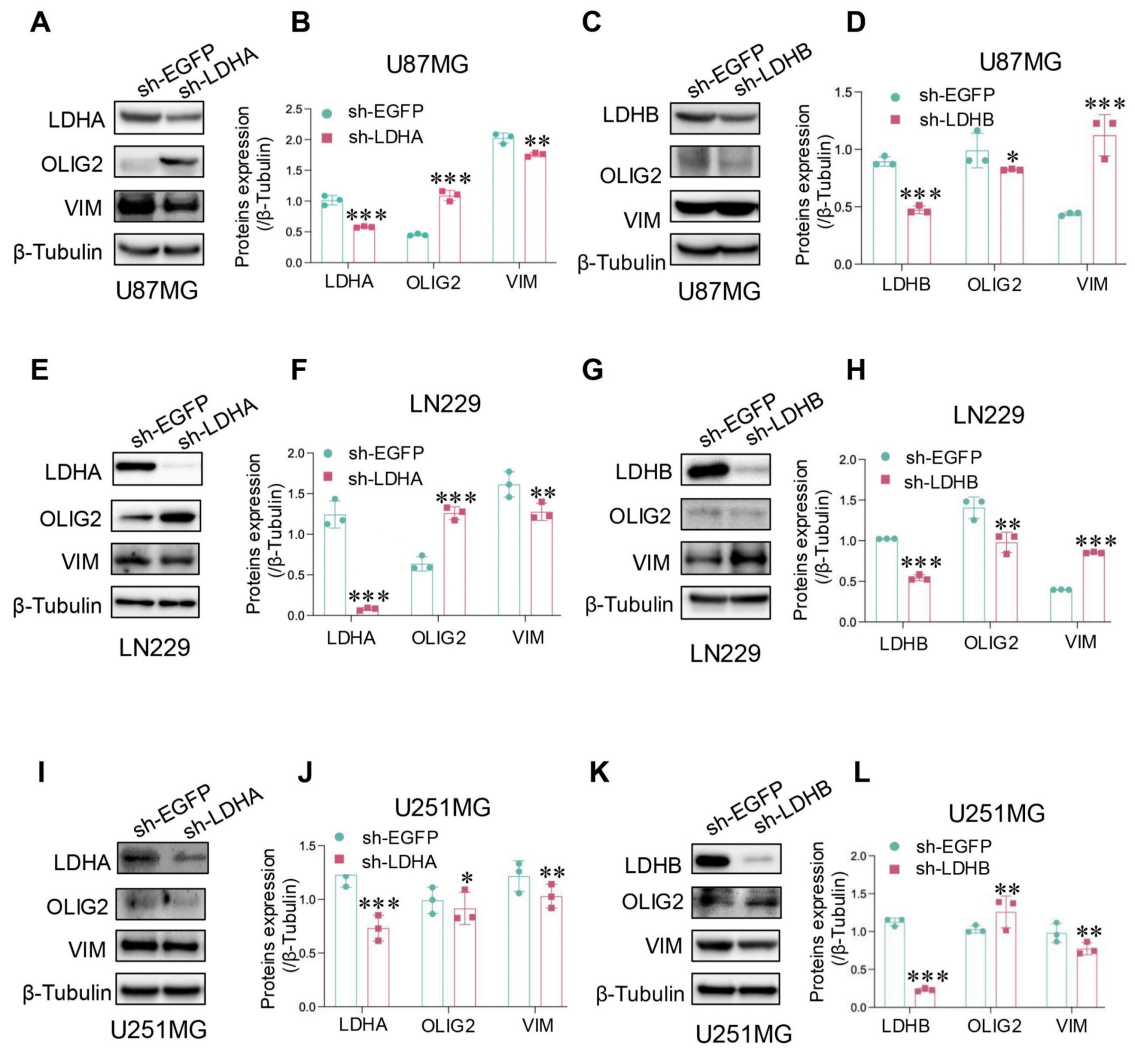
LDH isoenzymes promoted the PMT of glioma cells. (**A, B**) Protein expression of mesenchymal and proneural markers detected after LDHA knockdown in U87MG cells using western blotting and subsequent quantitative analysis (B). (**C, D**) Protein expression of mesenchymal and proneural markers detected after LDHB knockdown in U87MG cells using western blotting (C) and subsequent quantitative analysis (D). (**E, F**) Protein expression of mesenchymal and proneural markers detected after LDHA knockdown in LN229 cells, using western blotting (E) and subsequent quantitative analysis (F). (**G, H**) Protein expression of mesenchymal and proneural markers detected after LDHB knockdown in LN229 cells using western blotting (G) and subsequent quantitative analysis (H). (**I-L**) Detection of mesenchymal and proneural markers levels after LDHA (I) or LDHB (K) knockdown in U251MG cells, with quantification of the results (J, L). Data are presented as the mean ± standard deviation. Statistical analysis was conducted using one-way ANOVA; *P<0.05, **P<0.01, and ***P<0.001.

To further investigate the relationship between LDH isoenzymes and PMT in glioma, we analyzed protein expression levels of VIM (mesenchymal marker) and OLIG2 (proneural marker) through western blotting. The results confirmed that LDHA knockdown downregulated mesenchymal marker expression in U87MG cells, while proneural marker expression was upregulated (Fig. 5A and B). However, LDHB knockdown triggered the downregulation of the proneural marker and upregulation of mesenchymal markers (Fig. 5C and D). The corresponding findings in LN229 cells were consistent with these results (Fig. 5E-H), suggesting that LDH5 promotes PMT in mesenchymal glioma cells. Conversely, in U251MG cells, LDHA shRNA had a limited impact on mesenchymal transition, while treatment with LDHB shRNA decreased the expression of mesenchymal markers and increased that of proneural markers (Fig. 5I-L). Thus, LDH1 emerges as a pivotal factor driving PMT in proneural glioma cells. Given that the proneural subtype exhibits a higher propensity for transition into the mesenchymal subtype, it is imperative to intervene at the root of the process by targeting LDH1 in proneural subtype GBM.

### Synthesis of GAA-BSA nanoparticles for LDH1 treatment in proneural subtype GBM

**Figure 6:**
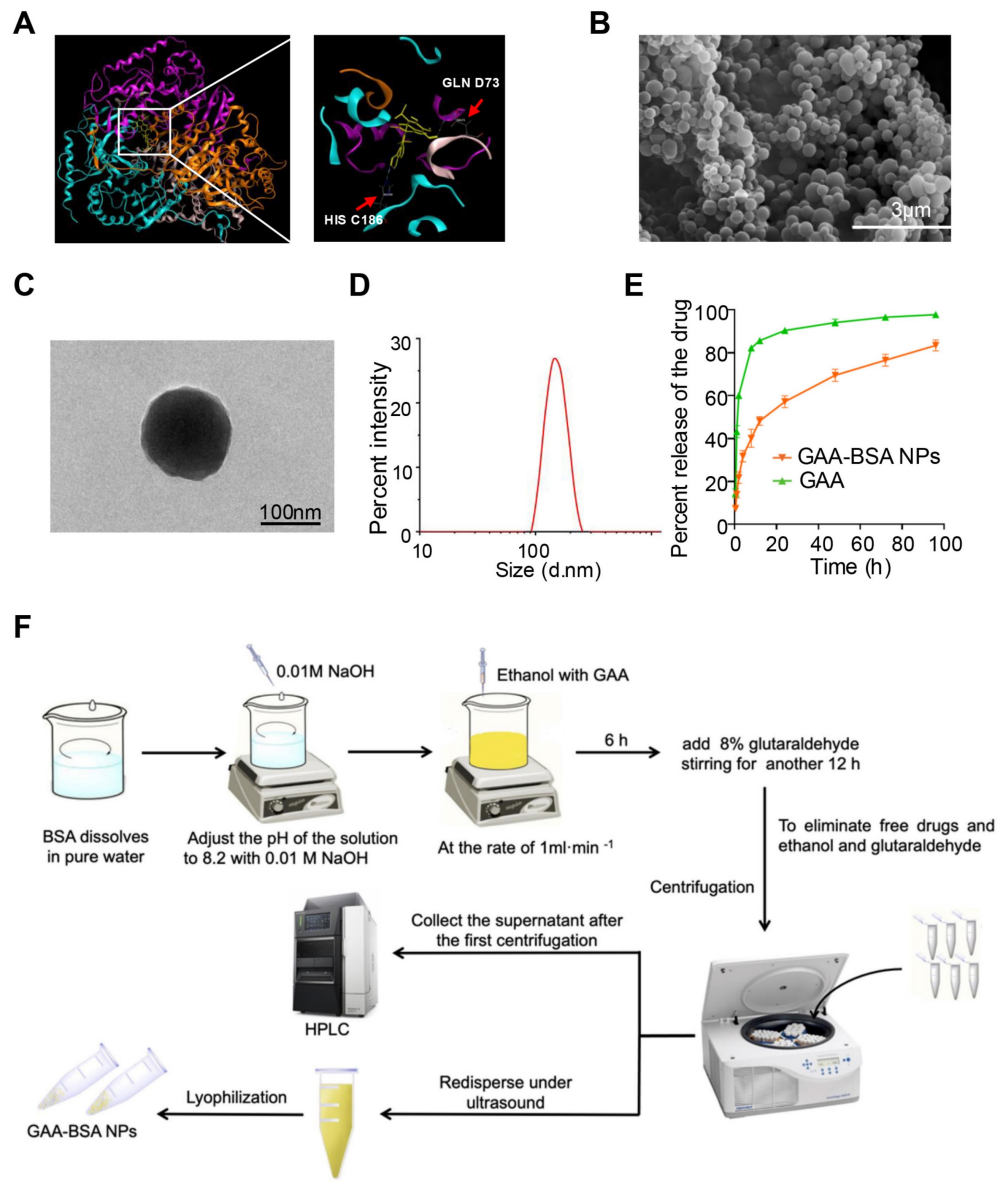
Preparation and characterization of GAA-BSA NPs. (**A**) Computational simulation depicting the binding sites of GAA on LDH1 protein, the arrows indicate the specific binding site GLN D73 and HIS C186. (**B**) Scanning electron microscopy (SEM) image illustrating GAA-BSA NPs (scale bar: 3 μm). (**C**) Transmission electron microscopy (TEM) image of GAA-BSA NPs (scale bar: 100 nm). (**D**) Particle size analysis of GAA-BSA NPs. (**E**) Comparative evaluation of *in vitro* drug release profiles between GAA solution and GAA-BSA NP formulation. (**F**) Schematic drawing of the preparation of GAA-BSA NPs.

Initially, a computational simulation was conducted to screen 10 drugs, revealing that gossypol-acetic acid (GAA) emerged as the most promising candidate for binding to the LDH1 protein. However, GAA exhibits poor water solubility and is prone to degradation in the tumor microenvironment 30. Bovine serum albumin (BSA), known for its exceptional biocompatibility and biodegradability, is a commonly utilized drug carrier 31. Notably, BSA nanoparticles are water-soluble, stable, and capable of slowly releasing hydrophobic drugs 32. Therefore, to mitigate the impact of the tumor microenvironment, we devised a strategy to encapsulate GAA within BSA protein nanoparticles (NPs), resulting in a novel formulation termed GAA-BSA nanoparticles. The binding sites of GAA on the LDH1 protein were simulated, revealing GLN D73 and HIS C186 as key interaction points (Fig. 6A). These results underscored the potential ability of GAA to target LDH1 protein. Subsequently, GAA-BSA NPs were synthesized and as shown in Fig. 6B and C, scanning electron microscopy (SEM) and transmission electron microscopy (TEM) imaging showed that GAA-BSA NPs were primarily homogenous in size and have a dense core. The average size of the NPs was 147.25±1.56 nm and the zeta potential was -28.87±0.52 mV (Fig. 6D; Table 2), which may prolonged the circulation time in the blood by increasing the blood-brain barrier permeability, targeting the clathrin-mediated endocytic pathway or evading phagocytosis by immune cells 33-36. Moreover, the NPs displayed a drug-loading efficiency (DLE) of 70.55±1.08%, with a drug-loading capacity (DLC) of 3.10±0.05% (Table 2), which is equivalent to 31.0 μg of GAA per mg of NPs. Drug release from the nanoparticles exhibited a biphasic pattern at pH 7.4, with an initial burst release of 31.78±2.17% within the first 4 h, followed by a gradual release of 83.36±2.0% over the subsequent 90 h (Fig. 6E). Figure 6F depicts a schematic of the preparation of GAA-BSA NPs using the desolvation method.

### GAA-BSA NPs inhibited proneural subtype GBM growth in vitro and in vivo

**Figure 7:**
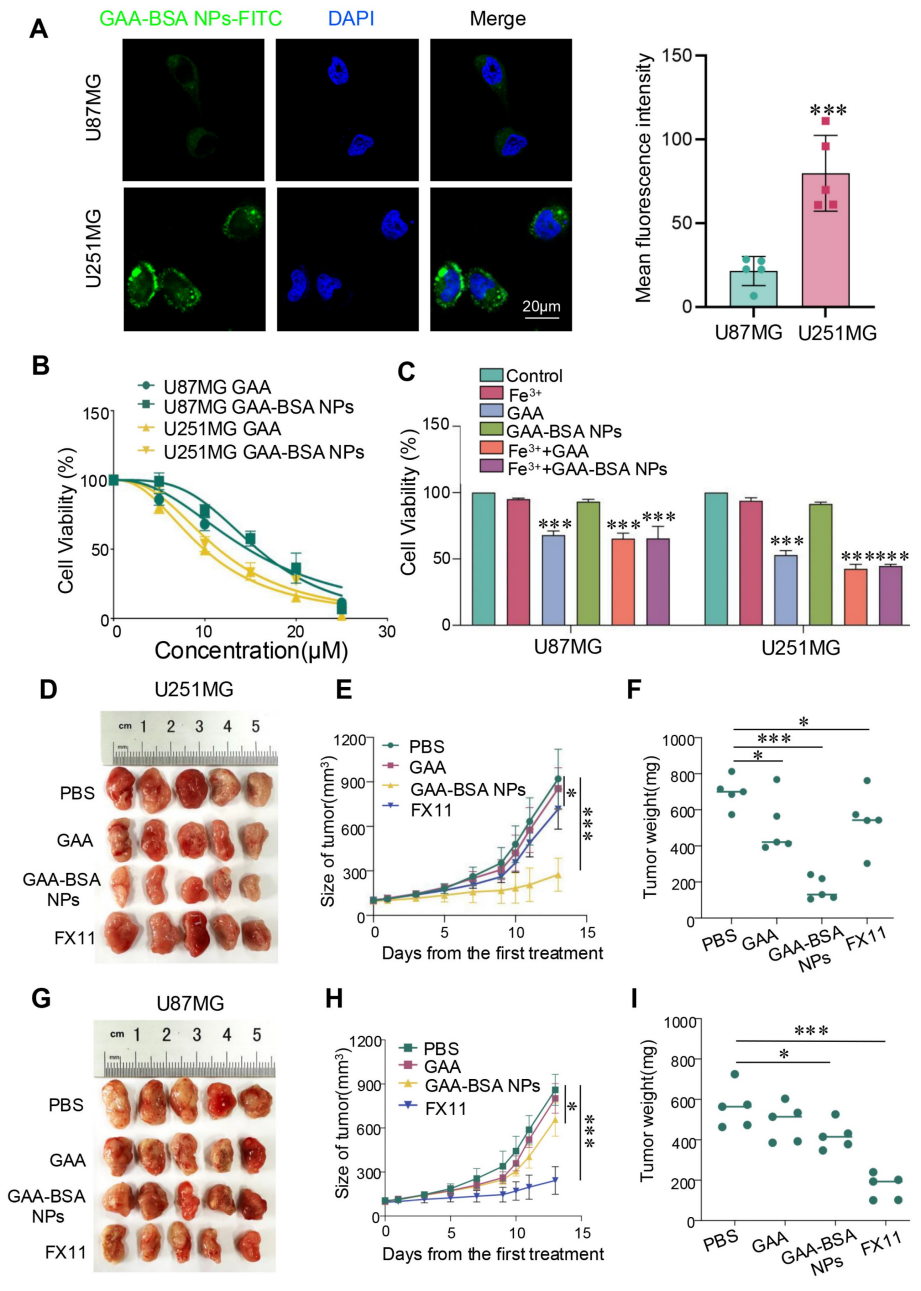
The therapeutic efficacy of GAA-BSA NPs for two subtypes of GBM *in vitro* and *in vivo*. (**A**) Visualization of U87MG and U251MG cells treated with GAA-BSA NPs. Cellular nuclei stained with DAPI (blue); intracellular FITC fluorescence (green); and merged images illustrating spatial overlap. Scale bar: 20 μm. (**B, C**) CCK-8 assay was used to detect the IC_50_ and cytotoxic potential of GAA and GAA-BSA NPs in U87MG and U251MG glioma cell lines (Fe^3+^: 50 μM Fecl_3_, GAA: 10 μM, GAA-BSA: 200 μg/mL including 10 μM GAA). (**D**) Photographic depiction of U251MG tumor samples post treatment. (**E**) Measurement of U251MG tumor volume progression over different time points. (**F**) Average tumor weights of U251MG tumors. (**G**) Photographs illustrating U87MG model tumors following treatment. (**H**) Alterations in tumor volume after treatment of U87MG model tumors with various regimens. (**I**) Mean tumor weights for U87MG model tumors. Mice in the control group were treated with PBS, while those in other groups received free GAA or GAA-BSA NPs, at a dose of 15 mg/kg of GAA; or LDHA inhibitor FX11 at 2.5 mg/kg. All data are presented as the mean ± standard deviation. Statistical analysis was conducted using one-way ANOVA; *P<0.05 and ***P<0.001.

The selective efficacy of GAA-BSA NPs was substantiated by observing cellular uptake in mesenchymal and proneural subtype cells. FITC-conjugated NPs were incubated with U87MG and U251MG cells for 4h. And significantly higher fluorescence intensity was observed in proneural subtype cells than in mesenchymal-type cells. (Fig. 7A). To verify the potential efficacy of GAA-BSA NPs in tumor treatment, *in vitro* cytotoxicity evaluation by CCK-8 assay was performed. The half-maximal inhibitory concentration (IC_50_) values of GAA against U87MG and U251MG cell lines were determined to be 14.73 and 9.91 μM, respectively. For GAA-BSA, the IC_50_ values were 290 μg/mL and 214.6 μg/mL, respectively (the GAA concentration was 15.79 and 11.50 μM, respectively). These results suggest substantial selectivity of GAA-BSA NPs for targeting and eliminating proneural subtype cells (Fig. 7B).

**Figure 8:**
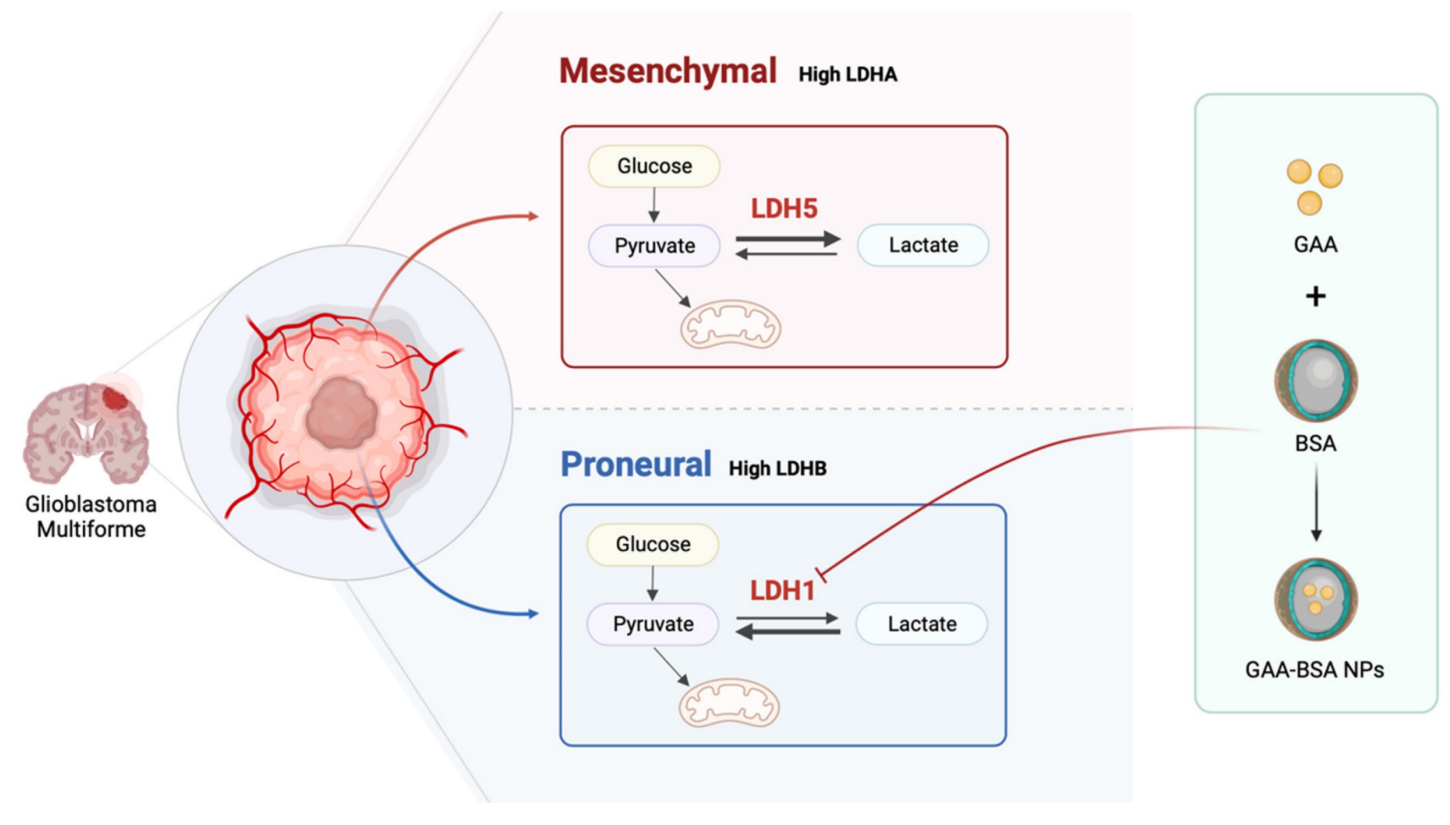
Schematic model showing the role of LDH isoenzyme in subtype classification between mesenchymal and proneural GBM. LDH5 was prominently involved in the mesenchymal subtype, while LDH1 was associated with the proneural subtype. Moreover, LDH1-targeting GAA-BSA NPs were recognized as a potential treatment approach for proneural-subtype GBM. The illustration was created using BioRender (https://www. biorender.com).

Importantly, this study assessed the potential of GAA-BSA NPs to neutralize the impact of Fe^3+^ in the tumor microenvironment. The CCK-8 assay results showed that cell viability was significantly reduced in the Fe^3+^+GAA-BSA NPs group compared to the Fe^3+^+GAA group. This observation indicated the capacity of GAA-BSA NPs to enhance cell killing by mitigating the effects of Fe^3+^ (Fig. 7C). To further assess the therapeutic efficacy of GAA-BSA NPs, we analyzed the tumor size, volume, and weight as indicators of treatment effect* in vivo*. FX11, an LDHA inhibitor, is utilized to assess the therapeutic sensitivity of mesenchymal and proneural subtype GBM. In proneural type tumor model, tumor size, volume, and weight were markedly reduced after the treatment with GAA-BSA NPs, surpassing the impact of GAA and LDHA inhibitor FX11 (Fig. 7D-F). However, this effect was not observed in the mesenchymal tumor model (Fig. 7G-I). In summary, GAA-BSA NPs may be a promising treatment for targeting proneural subtype GBM. The schematic model is shown in Fig. 8.

## Discussion

Although extensive studies have begun to unravel the connection between GBM molecular subtypes and metabolic phenotypes 37, the complex metabolic mechanisms driving subtype classification in GBM remain unclear. This study primarily identified LDH isoenzymes as correlating with mesenchymal and proneural subtype classification and demonstrated prognostic and clinical significance in GBM patients.

LDH is used as a diagnostic biomarker for various tumors and has a long history of application in routine clinical diagnosis 38. Recently, research attention has shifted to total serum LDH activity, as well as the expression of LDH isoenzymes 39. Our findings corroborate the notion that LDH isoenzymes might serve as clinically significant molecular markers for distinguishing mesenchymal and proneural GBM subtypes, offering a foundation for more precise molecular typing of GBM. The mesenchymal subtype, which is characterized by heightened aggressiveness and poor prognosis relative to the proneural subtype, can transition from the proneural subtype following radiation therapy and chemotherapy 40. PMT in GBM is aligned with increased proliferation and migration of cells 41, 42. This is consistent with previous evidence on the role of LDH isoenzymes in governing cellular growth and migration 43-45. In this study, we revealed the roles of LDH5 in promoting the proliferation, migration, and PMT of mesenchymal glioma cells, and showed that LDH1 sustains these functions in proneural glioma cells. The present evidence may serve as a cornerstone for developing gene expression-based diagnostic tools for recurrence monitoring and prognosis prediction. We hypothesized that targeting LDH isoenzymes could offer a potential strategy to inhibit this transition from the proneural subtype. This approach may lead to the development of effective therapeutic options against GBM.

Furthermore, targeted therapeutic strategies can be tailored based on LDH isoenzyme expression characteristics. The present investigation of GAA-BSA NPs offers a potentially selective approach to GBM therapy. BSA is a versatile protein system for drug delivery, that is non-toxic, non-immunogenic, biocompatible, and biodegradable 31, 46. Notably, paclitaxel-loaded human serum albumin NPs (Abraxane) received approval from the US Food and Drug Administration in 2005 for cancer chemotherapy 47, 48. In this context, we synthesized LDH1-targeting GAA-BSA NPs as a targeted therapeutic modality for proneural subtype GBM. These NPs were shown to effectively suppress proneural subtype tumor growth both *in vitro* and *in vivo*, with higher efficacy than against the mesenchymal subtype.

In conclusion, the results offer novel insights into the role of LDH isoenzyme in subtype classification between mesenchymal and proneural GBM and provide a promising treatment approach for proneural-subtype GBM.

## Supplementary Material

Supplementary figures.

## Figures and Tables

**Table 1:** Sequences of primers used in qRT-PCR

	Forward sequences	Reverse sequences
LDHA	5′-CCAACATGGCAGCCTTTTCC-3′	5′-TCACGTTACGCTGGACCAAA-3′
LDHB	5′-TGACTTTGTCTTCTCCGCACG -3′	5′-TCAGCCAGAGACTTTCCCAGA-3′
β-actin	5′-CACCATTGGCAATGAGCGGTTC-3′	5′-AGGTCTTTGCGGATGTCCACGT-3′

**Table 2:** Summary of the Characteristics of GAA-BSA NPs; data are shown as mean ± SD (n = 3)

Formulation	GAA-BSA
Particle size (nm)	147.25 ± 1.56
PDI	0.118 ± 0.021
Zeta-potential (mV)	-28.87 ± 0.52
DLE (%)	70.55 ± 1.08
DLC (%)	3.10 ± 0.05

## Abbreviations

LDH: lactate dehydrogenase
GAA: gossypol-acetic acid
BSA: bovine serum albumin
PMT: proneural-to-mesenchymal transition
DLC: drug-loading capacity
DLE: drug-loading efficiency
FBS: fetal bovine serum
PBS: phosphate-buffered saline
SEM: scanning electron microscopy
TCGA: the cancer genome atlas
