# Supplementary material for: LDH Isoenzyme and GAA-BSA Nanoparticles: A Novel Therapy Approach for Proneural Subtype Glioblastoma Multiforme
Source: J Cancer. 2025 Jan 6;16(4):1101–17. doi: 10.7150/jca.98452 (PMC11786042; doi:10.7150/jca.98452)
Supplement: Supplementary file 1 — Supplementary figures. [file jcav16p1101s1.pdf]

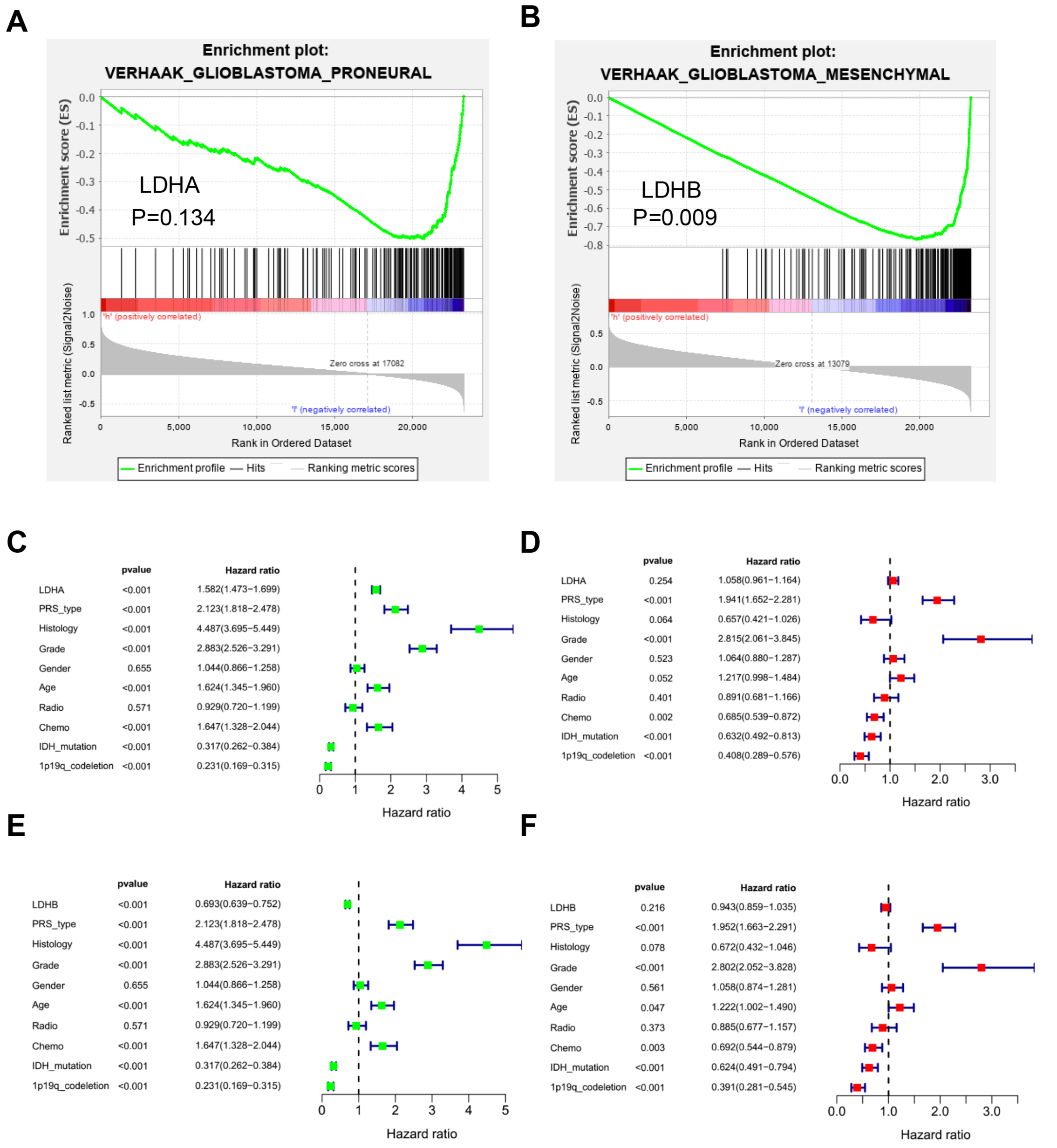

Figure S1 Prognostic and clinical significance of LDH subunits in GBM. (**A, B**) GSEA based on the Verhaak gene dataset illustrated that LDHA and LDHB expression was negatively correlated with the proneural (A) and mesenchymal (B) subtype, respectively (P=0.134 and 0.009, respectively). (**C, D**) Results of univariate (C) and multivariate (D) Cox proportional hazards regression analyses conducted for LDHA. (**E, F**) Results of univariate (E) and multivariate (F) Cox proportional hazards regression analyses

conducted for LDHB.

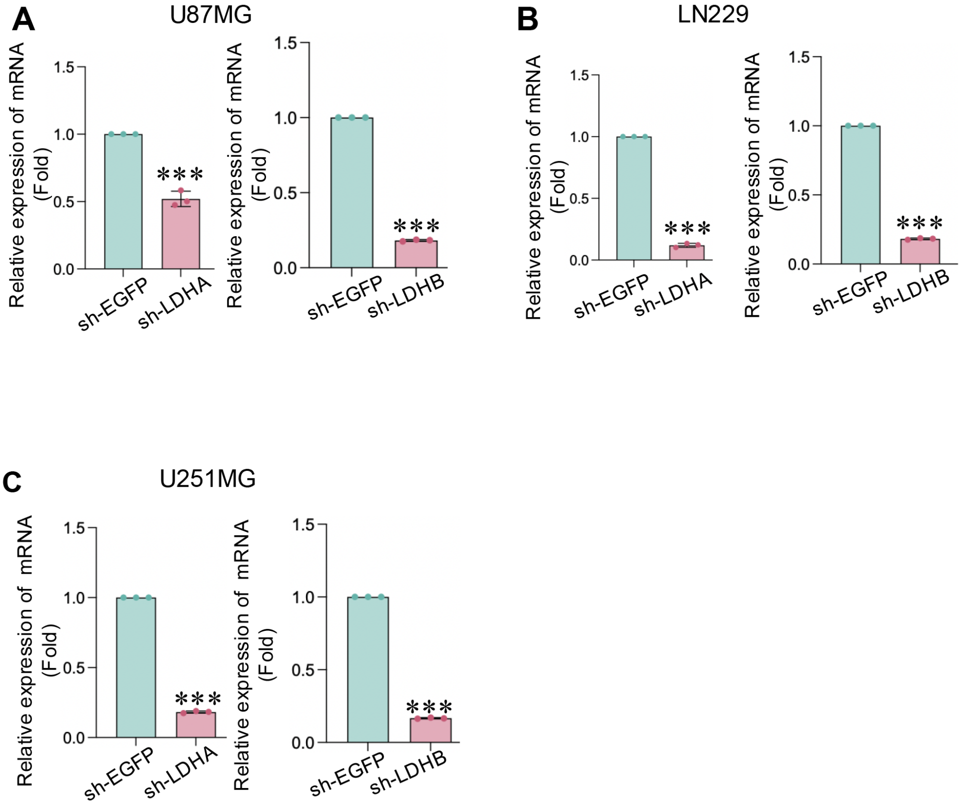

Figure S2 LDHA or LDHB knockdown in human glioma cells. (**A-C**) qRT-PCR demonstrating the levels of LDHA and LDHB expression in U87MG (A), LN229 (B) and U251MG (C) cells. Statistical analysis was conducted using one-way ANOVA; ***P<0.001.

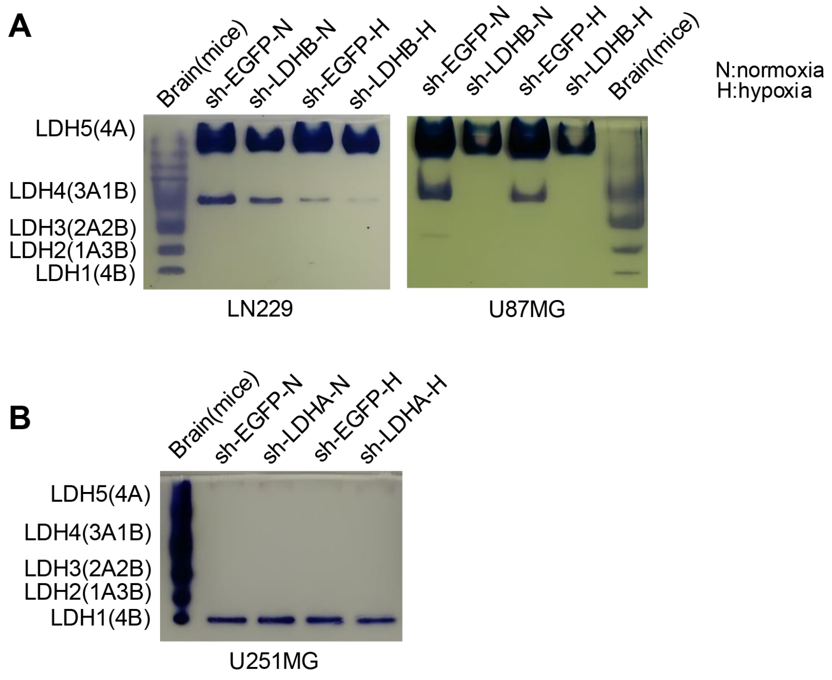

Figure S3 LDH isoenzyme expression of glioma cells. (**A, B**) Alterations resulting from LDHA or LDHB knockdown in LN229 and U87MG (A) and U251MG (B) cells.
